# From Takotsubo to Yamaguchi

**DOI:** 10.7759/cureus.23561

**Published:** 2022-03-28

**Authors:** Anoop Titus, Nitish Sharma, Gayatri Narayan, Yasar Sattar, Dimitrios Angelis

**Affiliations:** 1 Internal Medicine, Saint Vincent Hospital, Worcester, USA; 2 Cardiology, Saint Vincent Hospital, Worcester, USA; 3 Cardiology, West Virginia University, Morgantown, USA

**Keywords:** 2-dimensional echocardiography, angina, apical hypertrophy cardiomyopathy, yamaguchi cardiomyopathy, takotsubo cardioyopathy

## Abstract

Our patient was a 56-year-old Caucasian female who had 34 emergency department visits to our center with recurrent chest pain, of which eleven were of cardiac etiology, involving cardiac causes over the period of seven years. Her chest pain was diagnosed as atypical during her previous visits. Chest CT revealed “ace-of-spades” in the cardiac transverse section. A transthoracic echocardiogram (TTE) demonstrated apical hypertrophy with end-systolic cavity obliteration and an ejection fraction (EF) of 65%-70%, seated amidst a normal-sized left ventricle, with normal wall thickness, indicating Yamaguchi syndrome. In the case report, we portray the need to widen the spectrum of differentials in an encounter with a patient presenting with chest pain.

## Introduction

Apical hypertrophic cardiomyopathy (ApHCM) is a rare subset of hypertrophic cardiomyopathy that is characterized by a selectively thickened left ventricular (LV) apical segment to produce a “spade-shaped” cavity. It is most prevalent in Japan, amongst males, with outliers in other ethnic populations. The prevalence of ApHCM in males is higher despite ethnic origin. We present a case of recurrent chest pain and the evolution of diagnoses pertaining to the cardiac apex, spanning from Takotsubo cardiomyopathy (apical hypokinesis) to Yamaguchi syndrome (apical hypertrophy) in the same patient over a span of seven years [1-2].

## Case presentation

A 56-year-old Caucasian female with a past medical history of type 2 diabetes mellitus, chronic obstructive pulmonary disease, intravenous drug abuse, hypertension, hyperlipidemia, obstructive sleep apnea, adrenal insufficiency, and hepatitis C presented with chest pain, shortness of breath, and recurrent falls. During an admission seven years ago, she was diagnosed with Takotsubo cardiomyopathy, identified through cardiac catheterization, and was transiently on beta-blockers. Thereafter, she had multiple admissions with typical and atypical angina symptoms. Her last echocardiogram revealed normal to impaired LV function, with an ejection fraction (EF) of 60%-65%, and Holter was negative. The patient was re-admitted with chest pain 5/10 in severity, and radiation to the left arm. Her home medications included clonazepam, trazodone, Spiriva, albuterol, hydrocortisone, and gabapentin.

**Figure 1:**
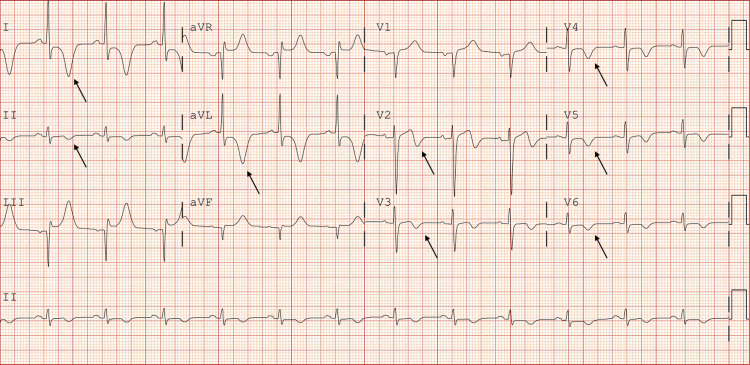
EKG demonstrating characteristic T-wave inversions with concerns for ischemia in leads I, II, aVL and V2-6

Initial vitals were normal. Electrocardiogram (EKG) showed deep inverted T-waves in I, II, aVL, and V2-V6 (Figure 1).

**Figure 2:**
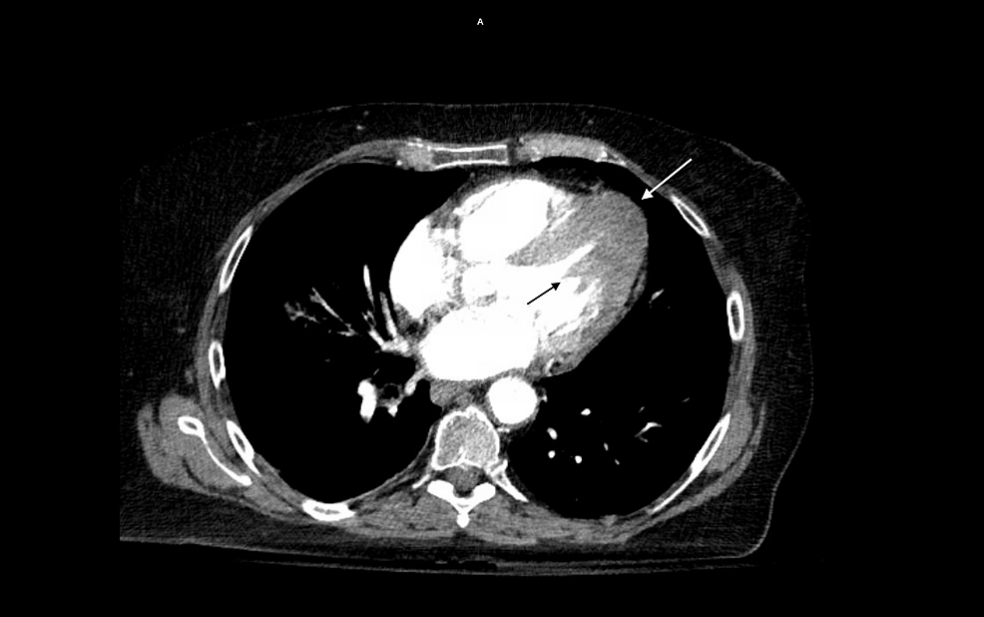
CT with contrast demonstrating apical hypertrophy and ace-of-spades appearance of the left ventricle

**Figure 3:**
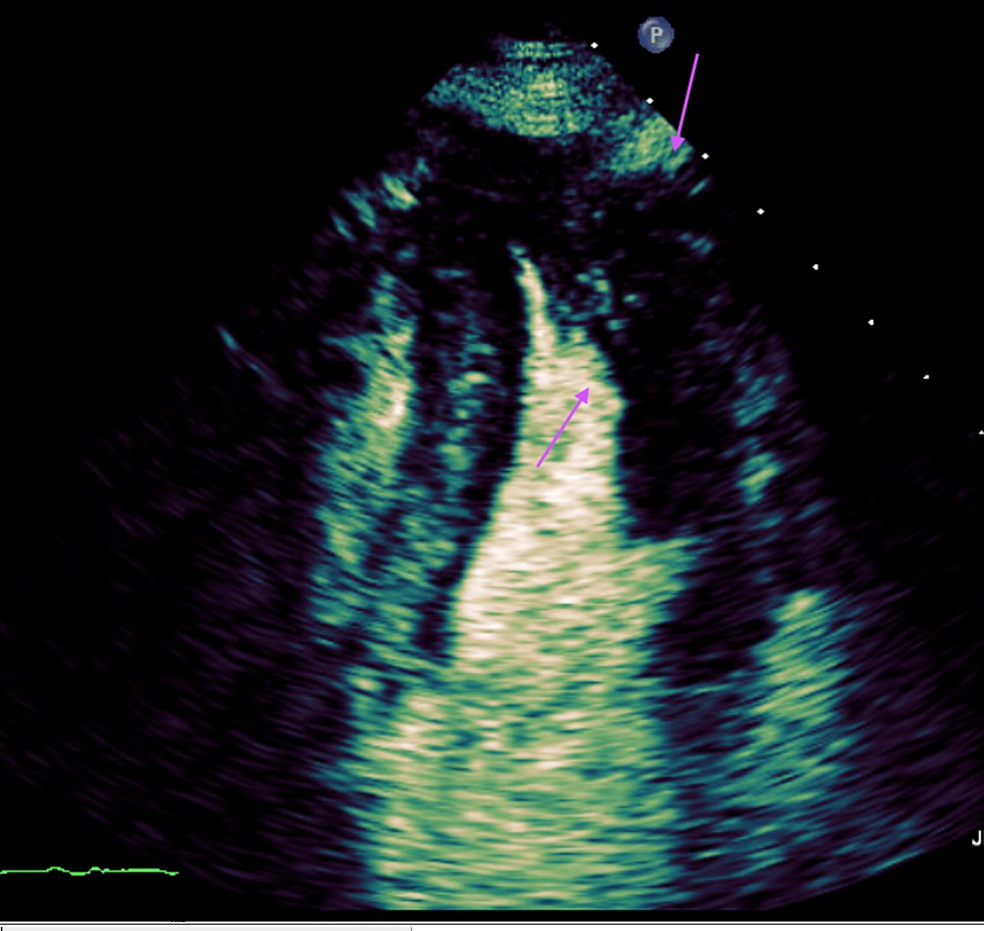
Transesophageal echocardiogram (TEE) with contrast demonstrating apical hypertrophy and end-diastolic obliteration with an ejection fraction (EF) of 65%-70%

Laboratory tests revealed troponins within normal limits and pro-brain natriuretic peptide (proBNP) of 2035 pg/ml; her chest X-ray was unremarkable. Chest CT revealed an “ace-of-spades” appearance in the cardiac transverse section (Figure 2). A transthoracic echocardiogram (TTE) demonstrated localized apical hypertrophy with end-systolic cavity obliteration and with an EF of 65%-70%, with a normal-sized left ventricle, normal wall thickness, indicating Yamaguchi syndrome (Figure 3). 

**Figure 4:**
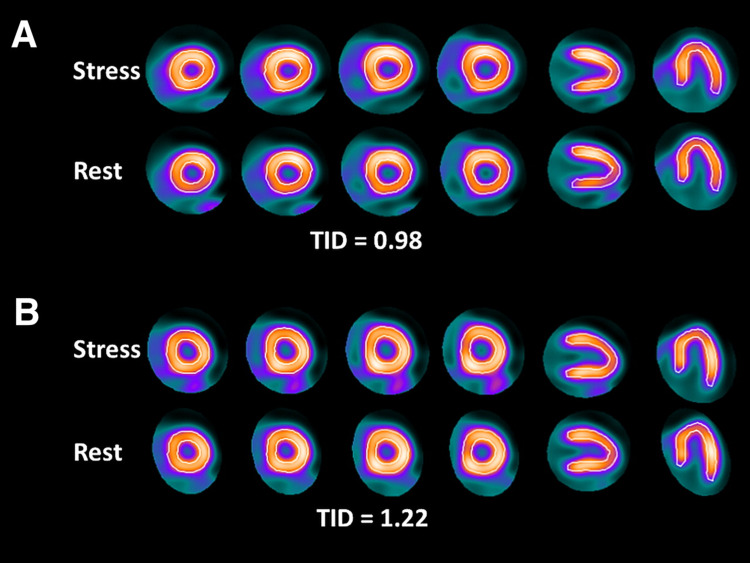
Myocardial perfusion scan demonstrating no evidence of ischemia TID: transient ischemic dilation.

Further workup with a non-invasive EKG stress test was negative for any obstructive coronary artery disease. The evaluation was then followed by a nuclear myocardial perfusion study which showed no evidence for ischemia or infarction (Figure 4). 

## Discussion

**Figure 5:**
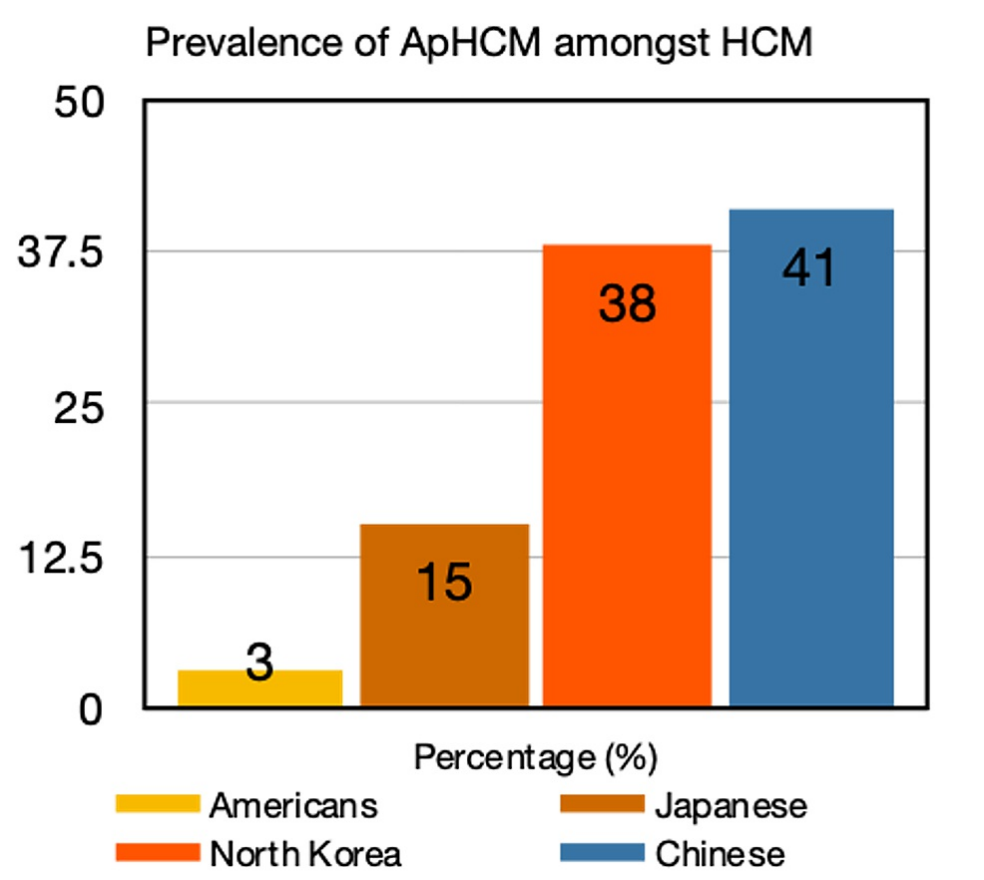
Graph depicting global prevalence of apical hypertrophic cardiomyopathy (ApHCM) amongst hypertrophic cardiomyopathy (HCM)

Hypertrophic cardiomyopathy, defined as the presence of a non-dilated left ventricular hypertrophy, in the absence of other cardiac, systemic, metabolic, or syndromic diseases, is seen in approximately 20 million people worldwide with the general population having a prevalence of 1:500 [1-2] (Figure 5). Morphologically, they can localize to an apical, concentric, lateral wall, and right ventricular form.

Classification of hypertrophic cardiomyopathy

1. Midcavity obstructive hypertrophic cardiomyopathy occurs when there is a left ventricular outflow tract (LVOT) obstruction due to approximation of the septal and lateral wall.

2. Midcavity obstruction with LV apical aneurysm occurs with fibrosis and scarring of the apical tissue. This predisposes patients to sudden cardiac deaths, thromboembolisms, and advanced congestive heart failure (CHF).

3. In ApHCM, LV thickening is seen only in the LV apex of ≥ 15 mm, or by a ratio of apical to the posterior wall thickness of ≥ 1.5 during end-diastole [3].

Genetics and histopathology

ApHCM is an autosomal dominant sarcomere protein gene mutation. Identifiable pathogenic gene mutations are seen in 13%-25% of patients. Common mutations include MYBPC3 and MYH711 [4]. ApHCM can exist with or without an apical aneurysm. Some forms develop a mid-ventricular obstruction with cavity obliteration (MVOCO) [3]. There has not been any identified association between the two diseases mentioned in our case, but speculations have been made about potential biochemical changes leading from one syndrome to the other.

Investigations

A hallmark finding of ApHCM is the presence of ‘giant-negative-T’ waves in the lateral leads along with high-voltage QRS-complexes. Investigations with cardiac MRI (CMR) have shown that those without T-wave inversions had hypertrophy in the distal-most location of the septum or apex. In cases with more diffuse apical involvement, the classic T wave inversions were seen [5].

Echocardiography

Echocardiography is useful in showing the typical ‘ace of spades’ appearance of LV. The conventional LV wall thickness of 15 mm or above is used in the diagnosis of ApHCM.

Nuclear Myocardial Perfusion imaging

Patients with ApHCM demonstrate a “solar polar” map pattern at rest, as well as relative apical flow reserve on the stress images, even in the absence of coronary artery disease (CAD).

CMR

CMR is useful in terms of diagnosis, prognosis, and guiding therapy as ApHCM can lead to thrombus formation, thus increasing the risk of thromboembolic stroke [6]. Fibrotic tissue, as seen on CMR, is a cause of ventricular tachyarrhythmias. The presence of late gadolinium enhancement of ≥15% of LV myocardium on CMR has shown to have an increased risk for sudden cardiac death [7]. Such patients would benefit from an automatic implantable cardioverter-defibrillator (AICD).

**Figure 6:**
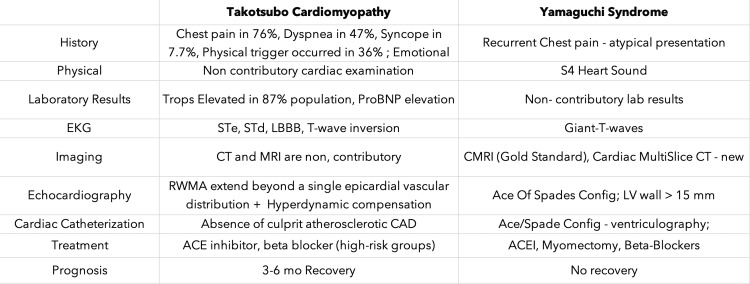
Difference between Takotsubo cardiomyopathy and Yamaguchi syndrome proBNP: pro-brain natriuretic peptide; STe: ST segment elevation; STd: ST segment depression; LBBB: left bundle branch block; RWMA: regional wall motion abnormalities; CMRI: cardiac magnetic resonance imaging; ACEI: angiotensin-converting enzyme inhibitors; CAD: coronary artery disease; LV: left ventricle.

The difference between Takotsubo cardiomyopathy and Yamaguchi syndrome is mentioned below (Figure 6).

Management

Management of ApHCM and other types of hypertrophic cardiomyopathy can be very challenging. ApHCM has no definite treatment. Usual treatment includes heart rate control and afterload reduction with atrioventricular nodal blockers and angiotensin-converting enzyme inhibitors [8]. Heart transplant and apical myomectomy are reserved for heart failure refractory to medical therapy [9].

Beta-blockers act by reducing the heart rate, improving the diastolic filling time, and reducing the mid-ventricular gradients. Anti-fibrotic properties of angiotensin-converting enzyme inhibitors (ACEI) and angiotensin II receptor blockers (ARBs) can be useful in ApHCM, given its pathophysiology of hypertrophy involved [10].

Only ~ 1% of hypertrophic cardiomyopathy patients undergo a cardiac transplant. Data on overall outcomes is limited. The overall transplant survival for hypertrophic cardiomyopathy patients at one, five, and ten years, was reported as 85%, 75%, and 61%, respectively. Septal ablation using alcohol has been used in hypertrophic cardiomyopathy. Its use in ApHCM is however limited. Apical myectomy is a newer procedure where the hypertrophic muscle from the apex and mid-left ventricle is excised. It was described by Schaff in 2010 using an apical approach [9]. The apical incision tends to produce akinetic areas. This has been reported to improve the functional status of patients.

There is a risk of thromboembolism in ApHCM with apical aneurysms, especially in those who have not been routinely anticoagulated. The choice of anticoagulation ranges from warfarin to novel oral anticoagulants. Aneurysms tend to be arrhythmogenic. A cardioverter-defibrillator can be implanted in such patients to prevent sudden cardiac death from life-threatening arrhythmias. Catheter ablation has not shown to be very helpful due to the technical difficulty of ablating a hypertrophied muscle and placing the catheter in the narrow neck of the aneurysm. Such cases deem surgical evaluation [11]. Newer drugs impacting diastolic function may play a role in the future. For example, ivabradine acts by inhibiting the pacing current, and perhexiline, an inhibitor of free fatty acid metabolism has recently been tried.

Our patient was treated with beta-blockers and an ACEI which resolved her symptoms in over a month.

## Conclusions

Our patient had multiple admissions in the past and demonstrated different diagnoses for her anginal presentations, ranging from Takotsubo cardiomyopathy to Yamaguchi syndrome. Although Yamaguchi syndrome is uncommon in Caucasians and females, our patient was considered low suspicion for the same until it was confirmed with investigations. This case intends to invoke insight into the possible differential diagnoses regarding trivial chest pain. It also throws light on the pathologies of the cardiac apex.
